# Supplementary material for: A fault-intrusion-tolerant system and deadline-aware algorithm for scheduling scientific workflow in the cloud
Source: PeerJ Comput Sci. 2021 Nov 2;7:e747. doi: 10.7717/peerj-cs.747 (PMC8576554; doi:10.7717/peerj-cs.747)
Supplement: Supplemental Information 6 [file peerj-cs-07-747-s006.zip › Myproject1.1/docs/org/cloudbus/cloudsim/Datacenter.html]

Datacenter


JavaScript is disabled on your browser.


- Overview
- Package
- Class
- Use
- Tree
- Deprecated
- Index
- Help

- Prev Class
- Next Class

- Frames
- No Frames

- All Classes

- Summary:
- Nested |
- Field |
- Constr |
- Method

- Detail:
- Field |
- Constr |
- Method


org.cloudbus.cloudsim

## Class Datacenter

- java.lang.Object
- - org.cloudbus.cloudsim.core.SimEntity
  - - org.cloudbus.cloudsim.Datacenter

- All Implemented Interfaces:
  :   java.lang.Cloneable

  Direct Known Subclasses:
  :   DatacenterExtended, NetworkDatacenter, PowerDatacenter

  ---

    

  ```
  public class Datacenter
  extends SimEntity
  ```

  Datacenter class is a CloudResource whose hostList are virtualized. It deals with processing of
  VM queries (i.e., handling of VMs) instead of processing Cloudlet-related queries. So, even
  though an AllocPolicy will be instantiated (in the init() method of the superclass, it will not
  be used, as processing of cloudlets are handled by the CloudletScheduler and processing of
  VirtualMachines are handled by the VmAllocationPolicy.

  Since:
  :   CloudSim Toolkit 1.0

- - ### Field Summary

    - ### Fields inherited from class org.cloudbus.cloudsim.core.SimEntity

      `FINISHED, HOLDING, RUNNABLE, WAITING`
  - ### Constructor Summary

    Constructors

    Constructor and Description

    `Datacenter(java.lang.String name,
    DatacenterCharacteristics characteristics,
    VmAllocationPolicy vmAllocationPolicy,
    java.util.List<Storage> storageList,
    double schedulingInterval)`

    Allocates a new PowerDatacenter object.
  - ### Method Summary

    Methods

    | Modifier and Type | Method and Description |
    | --- | --- |
    | `int` | `addFile(File file)` Adds a file into the resource's storage before the experiment starts. |
    | `protected void` | `checkCloudletCompletion()` Verifies if some cloudlet inside this PowerDatacenter already finished. |
    | `protected boolean` | `contains(File file)` Checks whether the resource has the given file. |
    | `protected boolean` | `contains(java.lang.String fileName)` Checks whether the resource has the given file. |
    | `protected DatacenterCharacteristics` | `getCharacteristics()` Gets the characteristics. |
    | `<T extends Host>  java.util.List<T>` | `getHostList()` Gets the host list. |
    | `protected double` | `getLastProcessTime()` Gets the last process time. |
    | `protected java.lang.String` | `getRegionalCisName()` Gets the regional cis name. |
    | `protected double` | `getSchedulingInterval()` Gets the scheduling interval. |
    | `protected java.util.List<Storage>` | `getStorageList()` Gets the storage list. |
    | `VmAllocationPolicy` | `getVmAllocationPolicy()` Gets the vm allocation policy. |
    | `<T extends Vm>  java.util.List<T>` | `getVmList()` Gets the vm list. |
    | `protected double` | `predictFileTransferTime(java.util.List<java.lang.String> requiredFiles)` Predict file transfer time. |
    | `protected void` | `processCloudlet(SimEvent ev, int type)` Processes a Cloudlet based on the event type. |
    | `protected void` | `processCloudletCancel(int cloudletId, int userId, int vmId)` Processes a Cloudlet cancel request. |
    | `protected void` | `processCloudletMove(int[] receivedData, int type)` Process the event for an User/Broker who wants to move a Cloudlet. |
    | `protected void` | `processCloudletPause(int cloudletId, int userId, int vmId, boolean ack)` Processes a Cloudlet pause request. |
    | `protected void` | `processCloudletResume(int cloudletId, int userId, int vmId, boolean ack)` Processes a Cloudlet resume request. |
    | `protected void` | `processCloudletStatus(SimEvent ev)` Process the event for an User/Broker who wants to know the status of a Cloudlet. |
    | `protected void` | `processCloudletSubmit(SimEvent ev, boolean ack)` Processes a Cloudlet submission. |
    | `protected void` | `processDataAdd(SimEvent ev, boolean ack)` Process data add. |
    | `protected void` | `processDataDelete(SimEvent ev, boolean ack)` Process data del. |
    | `void` | `processEvent(SimEvent ev)` Processes events or services that are available for this PowerDatacenter. |
    | `protected void` | `processOtherEvent(SimEvent ev)` Here all the method related to VM requests will be received and forwarded to the related method. |
    | `protected void` | `processPingRequest(SimEvent ev)` Processes a ping request. |
    | `protected void` | `processVmCreate(SimEvent ev, boolean ack)` Process the event for an User/Broker who wants to create a VM in this PowerDatacenter. |
    | `protected void` | `processVmDestroy(SimEvent ev, boolean ack)` Process the event for an User/Broker who wants to destroy a VM previously created in this PowerDatacenter. |
    | `protected void` | `processVmMigrate(SimEvent ev, boolean ack)` Process the event for an User/Broker who wants to migrate a VM. |
    | `protected void` | `registerOtherEntity()` Overrides this method when making a new and different type of resource. |
    | `protected void` | `setCharacteristics(DatacenterCharacteristics characteristics)` Sets the characteristics. |
    | `protected void` | `setLastProcessTime(double lastProcessTime)` Sets the last process time. |
    | `protected void` | `setRegionalCisName(java.lang.String regionalCisName)` Sets the regional cis name. |
    | `protected void` | `setSchedulingInterval(double schedulingInterval)` Sets the scheduling interval. |
    | `protected void` | `setStorageList(java.util.List<Storage> storageList)` Sets the storage list. |
    | `protected void` | `setVmAllocationPolicy(VmAllocationPolicy vmAllocationPolicy)` Sets the vm allocation policy. |
    | `protected <T extends Vm>  void` | `setVmList(java.util.List<T> vmList)` Sets the vm list. |
    | `void` | `shutdownEntity()` This method is invoked by the `Simulation` before the simulation finishes. |
    | `void` | `startEntity()` This method is invoked by the `Simulation` class when the simulation is started. |
    | `protected void` | `updateCloudletProcessing()` Updates processing of each cloudlet running in this PowerDatacenter. |

    - ### Methods inherited from class org.cloudbus.cloudsim.core.SimEntity

      `cancelEvent, clone, getEventBuffer, getId, getName, getNextEvent, getNextEvent, getState, numEventsWaiting, numEventsWaiting, pause, run, schedule, schedule, schedule, schedule, scheduleFirst, scheduleFirst, scheduleFirst, scheduleFirst, scheduleFirstNow, scheduleFirstNow, scheduleFirstNow, scheduleFirstNow, scheduleNow, scheduleNow, scheduleNow, scheduleNow, selectEvent, send, send, send, send, sendNow, sendNow, sendNow, sendNow, setEventBuffer, setId, setState, waitForEvent`
    - ### Methods inherited from class java.lang.Object

      `equals, finalize, getClass, hashCode, notify, notifyAll, toString, wait, wait, wait`

- - ### Constructor Detail


    - #### Datacenter

      ```
      public Datacenter(java.lang.String name,
                DatacenterCharacteristics characteristics,
                VmAllocationPolicy vmAllocationPolicy,
                java.util.List<Storage> storageList,
                double schedulingInterval)
                 throws java.lang.Exception
      ```

      Allocates a new PowerDatacenter object.

      Parameters:
      :   `name` - the name to be associated with this entity (as required by Sim\_entity class from
          simjava package)
      :   `characteristics` - an object of DatacenterCharacteristics
      :   `storageList` - a LinkedList of storage elements, for data simulation
      :   `vmAllocationPolicy` - the vmAllocationPolicy

      Throws:
      :   `java.lang.Exception` - This happens when one of the following scenarios occur:
          - creating this entity before initializing CloudSim package- this entity name is null or empty- this entity has zero number of PEs (Processing Elements).   
                No PEs mean the Cloudlets can't be processed. A CloudResource must contain one or
                more Machines. A Machine must contain one or more PEs.
  - ### Method Detail


    - #### registerOtherEntity

      ```
      protected void registerOtherEntity()
      ```

      Overrides this method when making a new and different type of resource.   
      **NOTE:** You do not need to override `#body()` method, if you use this method.


    - #### processEvent

      ```
      public void processEvent(SimEvent ev)
      ```

      Processes events or services that are available for this PowerDatacenter.

      **Specified by:**
      :   `processEvent` in class `SimEntity`

      Parameters:
      :   `ev` - a Sim\_event object


    - #### processDataDelete

      ```
      protected void processDataDelete(SimEvent ev,
                           boolean ack)
      ```

      Process data del.

      Parameters:
      :   `ev` - the ev
      :   `ack` - the ack


    - #### processDataAdd

      ```
      protected void processDataAdd(SimEvent ev,
                        boolean ack)
      ```

      Process data add.

      Parameters:
      :   `ev` - the ev
      :   `ack` - the ack


    - #### processPingRequest

      ```
      protected void processPingRequest(SimEvent ev)
      ```

      Processes a ping request.

      Parameters:
      :   `ev` - a Sim\_event object


    - #### processCloudletStatus

      ```
      protected void processCloudletStatus(SimEvent ev)
      ```

      Process the event for an User/Broker who wants to know the status of a Cloudlet. This
      PowerDatacenter will then send the status back to the User/Broker.

      Parameters:
      :   `ev` - a Sim\_event object


    - #### processOtherEvent

      ```
      protected void processOtherEvent(SimEvent ev)
      ```

      Here all the method related to VM requests will be received and forwarded to the related
      method.

      Parameters:
      :   `ev` - the received event


    - #### processVmCreate

      ```
      protected void processVmCreate(SimEvent ev,
                         boolean ack)
      ```

      Process the event for an User/Broker who wants to create a VM in this PowerDatacenter. This
      PowerDatacenter will then send the status back to the User/Broker.

      Parameters:
      :   `ev` - a Sim\_event object
      :   `ack` - the ack


    - #### processVmDestroy

      ```
      protected void processVmDestroy(SimEvent ev,
                          boolean ack)
      ```

      Process the event for an User/Broker who wants to destroy a VM previously created in this
      PowerDatacenter. This PowerDatacenter may send, upon request, the status back to the
      User/Broker.

      Parameters:
      :   `ev` - a Sim\_event object
      :   `ack` - the ack


    - #### processVmMigrate

      ```
      protected void processVmMigrate(SimEvent ev,
                          boolean ack)
      ```

      Process the event for an User/Broker who wants to migrate a VM. This PowerDatacenter will
      then send the status back to the User/Broker.

      Parameters:
      :   `ev` - a Sim\_event object


    - #### processCloudlet

      ```
      protected void processCloudlet(SimEvent ev,
                         int type)
      ```

      Processes a Cloudlet based on the event type.

      Parameters:
      :   `ev` - a Sim\_event object
      :   `type` - event type


    - #### processCloudletMove

      ```
      protected void processCloudletMove(int[] receivedData,
                             int type)
      ```

      Process the event for an User/Broker who wants to move a Cloudlet.

      Parameters:
      :   `receivedData` - information about the migration
      :   `type` - event tag


    - #### processCloudletSubmit

      ```
      protected void processCloudletSubmit(SimEvent ev,
                               boolean ack)
      ```

      Processes a Cloudlet submission.

      Parameters:
      :   `ev` - a SimEvent object
      :   `ack` - an acknowledgement


    - #### predictFileTransferTime

      ```
      protected double predictFileTransferTime(java.util.List<java.lang.String> requiredFiles)
      ```

      Predict file transfer time.

      Parameters:
      :   `requiredFiles` - the required files

      Returns:
      :   the double


    - #### processCloudletResume

      ```
      protected void processCloudletResume(int cloudletId,
                               int userId,
                               int vmId,
                               boolean ack)
      ```

      Processes a Cloudlet resume request.

      Parameters:
      :   `cloudletId` - resuming cloudlet ID
      :   `userId` - ID of the cloudlet's owner
      :   `ack` - $true if an ack is requested after operation
      :   `vmId` - the vm id


    - #### processCloudletPause

      ```
      protected void processCloudletPause(int cloudletId,
                              int userId,
                              int vmId,
                              boolean ack)
      ```

      Processes a Cloudlet pause request.

      Parameters:
      :   `cloudletId` - resuming cloudlet ID
      :   `userId` - ID of the cloudlet's owner
      :   `ack` - $true if an ack is requested after operation
      :   `vmId` - the vm id


    - #### processCloudletCancel

      ```
      protected void processCloudletCancel(int cloudletId,
                               int userId,
                               int vmId)
      ```

      Processes a Cloudlet cancel request.

      Parameters:
      :   `cloudletId` - resuming cloudlet ID
      :   `userId` - ID of the cloudlet's owner
      :   `vmId` - the vm id


    - #### updateCloudletProcessing

      ```
      protected void updateCloudletProcessing()
      ```

      Updates processing of each cloudlet running in this PowerDatacenter. It is necessary because
      Hosts and VirtualMachines are simple objects, not entities. So, they don't receive events and
      updating cloudlets inside them must be called from the outside.


    - #### checkCloudletCompletion

      ```
      protected void checkCloudletCompletion()
      ```

      Verifies if some cloudlet inside this PowerDatacenter already finished. If yes, send it to
      the User/Broker


    - #### addFile

      ```
      public int addFile(File file)
      ```

      Adds a file into the resource's storage before the experiment starts. If the file is a master
      file, then it will be registered to the RC when the experiment begins.

      Parameters:
      :   `file` - a DataCloud file

      Returns:
      :   a tag number denoting whether this operation is a success or not


    - #### contains

      ```
      protected boolean contains(File file)
      ```

      Checks whether the resource has the given file.

      Parameters:
      :   `file` - a file to be searched

      Returns:
      :   true if successful, false otherwise


    - #### contains

      ```
      protected boolean contains(java.lang.String fileName)
      ```

      Checks whether the resource has the given file.

      Parameters:
      :   `fileName` - a file name to be searched

      Returns:
      :   true if successful, false otherwise


    - #### shutdownEntity

      ```
      public void shutdownEntity()
      ```

      **Description copied from class: `SimEntity`**

      This method is invoked by the `Simulation` before the simulation finishes. If you want
      to save data in log files this is the method in which the corresponding code would be placed.

      **Specified by:**
      :   `shutdownEntity` in class `SimEntity`


    - #### startEntity

      ```
      public void startEntity()
      ```

      **Description copied from class: `SimEntity`**

      This method is invoked by the `Simulation` class when the simulation is started. This
      method should be responsible for starting the entity up.

      **Specified by:**
      :   `startEntity` in class `SimEntity`


    - #### getHostList

      ```
      public <T extends Host> java.util.List<T> getHostList()
      ```

      Gets the host list.

      Returns:
      :   the host list


    - #### getCharacteristics

      ```
      protected DatacenterCharacteristics getCharacteristics()
      ```

      Gets the characteristics.

      Returns:
      :   the characteristics


    - #### setCharacteristics

      ```
      protected void setCharacteristics(DatacenterCharacteristics characteristics)
      ```

      Sets the characteristics.

      Parameters:
      :   `characteristics` - the new characteristics


    - #### getRegionalCisName

      ```
      protected java.lang.String getRegionalCisName()
      ```

      Gets the regional cis name.

      Returns:
      :   the regional cis name


    - #### setRegionalCisName

      ```
      protected void setRegionalCisName(java.lang.String regionalCisName)
      ```

      Sets the regional cis name.

      Parameters:
      :   `regionalCisName` - the new regional cis name


    - #### getVmAllocationPolicy

      ```
      public VmAllocationPolicy getVmAllocationPolicy()
      ```

      Gets the vm allocation policy.

      Returns:
      :   the vm allocation policy


    - #### setVmAllocationPolicy

      ```
      protected void setVmAllocationPolicy(VmAllocationPolicy vmAllocationPolicy)
      ```

      Sets the vm allocation policy.

      Parameters:
      :   `vmAllocationPolicy` - the new vm allocation policy


    - #### getLastProcessTime

      ```
      protected double getLastProcessTime()
      ```

      Gets the last process time.

      Returns:
      :   the last process time


    - #### setLastProcessTime

      ```
      protected void setLastProcessTime(double lastProcessTime)
      ```

      Sets the last process time.

      Parameters:
      :   `lastProcessTime` - the new last process time


    - #### getStorageList

      ```
      protected java.util.List<Storage> getStorageList()
      ```

      Gets the storage list.

      Returns:
      :   the storage list


    - #### setStorageList

      ```
      protected void setStorageList(java.util.List<Storage> storageList)
      ```

      Sets the storage list.

      Parameters:
      :   `storageList` - the new storage list


    - #### getVmList

      ```
      public <T extends Vm> java.util.List<T> getVmList()
      ```

      Gets the vm list.

      Returns:
      :   the vm list


    - #### setVmList

      ```
      protected <T extends Vm> void setVmList(java.util.List<T> vmList)
      ```

      Sets the vm list.

      Parameters:
      :   `vmList` - the new vm list


    - #### getSchedulingInterval

      ```
      protected double getSchedulingInterval()
      ```

      Gets the scheduling interval.

      Returns:
      :   the scheduling interval


    - #### setSchedulingInterval

      ```
      protected void setSchedulingInterval(double schedulingInterval)
      ```

      Sets the scheduling interval.

      Parameters:
      :   `schedulingInterval` - the new scheduling interval


- Overview
- Package
- Class
- Use
- Tree
- Deprecated
- Index
- Help

- Prev Class
- Next Class

- Frames
- No Frames

- All Classes

- Summary:
- Nested |
- Field |
- Constr |
- Method

- Detail:
- Field |
- Constr |
- Method
